# Looking for Landmarks: The Role of Expert Review and Bibliometric Analysis in Evaluating Scientific Publication Outputs

**DOI:** 10.1371/journal.pone.0005910

**Published:** 2009-06-18

**Authors:** Liz Allen, Ceri Jones, Kevin Dolby, David Lynn, Mark Walport

**Affiliations:** Wellcome Trust, London, United Kingdom; Johns Hopkins Bloomberg School of Public Health, United States of America

## Abstract

**Objective:**

To compare expert assessment with bibliometric indicators as tools to assess the quality and importance of scientific research papers.

**Methods and Materials:**

Shortly after their publication in 2005, the quality and importance of a cohort of nearly 700 Wellcome Trust (WT) associated research papers were assessed by expert reviewers; each paper was reviewed by two WT expert reviewers. After 3 years, we compared this initial assessment with other measures of paper impact.

**Results:**

Shortly after publication, 62 (9%) of the 687 research papers were determined to describe at least a ‘major addition to knowledge’ –6 were thought to be ‘landmark’ papers. At an aggregate level, after 3 years, there was a strong positive association between expert assessment and impact as measured by number of citations and F1000 rating. However, there were some important exceptions indicating that bibliometric measures may not be sufficient in isolation as measures of research quality and importance, and especially not for assessing single papers or small groups of research publications.

**Conclusion:**

When attempting to assess the quality and importance of research papers, we found that sole reliance on bibliometric indicators would have led us to miss papers containing important results as judged by expert review. In particular, some papers that were highly rated by experts were not highly cited during the first three years after publication. Tools that *link* expert peer reviews of research paper quality and importance to more quantitative indicators, such as citation analysis would be valuable additions to the field of research assessment and evaluation.

## Introduction

The Wellcome Trust has spent over £2.5 bn on biomedical research during the last 5 years in pursuit of its mission ‘*to foster and promote research with the aim of improving human and animal health.*’ At any one time, we support over 3000 researchers in more than 50 countries. One of the major challenges we face is the evaluation of how and where our support is making a difference.

Like other organisations in the business of supporting research to generate new knowledge, while we recognise the array of outputs and impacts of research, the production of a scientific research paper remains a good indication of research progression and knowledge generation. There is a range of measures that can be used to indicate the value of a research paper. There is also a wide-ranging critique of bibliometric indicators [1], [2], [3]; knowing who has published what and where, and understanding how this work has been cited does not necessarily reflect the quality and importance of the research being described, the potential impacts or the longevity of a line of research. As Eugene Garfield wrote in a recent review of the history and uses of Journal Impact Factors, ‘*In an ideal world, evaluators would read each article and make personal judgements*’ [4]. It is this reasoning that has led to the development of more qualitative tools of assessment of published outputs, exemplified by the Faculty of 1000 (F1000) – an online service where a selected group (‘Faculty’) of leading researchers and clinicians highlight and evaluate what they consider to be the most important articles emerging in biology and medicine (http://www.facultyof1000.com/).

The emergence of more accessible electronic and web-based bibliographic and reference tools has greatly improved access to publication outputs. Since May 2005, the U.S. National Library of Medicine (NLM) has been identifying and indexing biomedical research papers, published in peer-reviewed journals and appearing on PubMed, where the Wellcome Trust has been cited in the acknowledgment section. From January 2008, this service has extended to all UK PubMed Central (UKPMC) funders. This presents unprecedented access to up-to-date, ‘live’ information on the funding sources for scientific published outputs and the opportunity to better understand the nature of those outputs.

We analysed the first 1000 research papers acknowledging Wellcome Trust funding, indexed on the PubMed database (published between May and September 2005) and tracked these for three years to measure how often the papers were cited. The project had three aims. The first was to characterise the spread of publications and to assess the ‘quality’ of a large cohort of Wellcome Trust associated publications, which prior to the PubMed indexing of ‘Wellcome Trust’ had been difficult to do. Our second aim was to compare the relative importance of publication outputs associated with different funding mechanisms – notably whether research funded through larger, longer-term grants yielded higher quality output than smaller, short-term grants - which is one of the most debated issues among research funders. Our third aim was to explore the extent to which expert predictions of ‘importance’ at the time of publication correlated with impact and use according to more traditional bibliometric measures. Comparison of expert review of scientific papers at the time of publication with subsequent citation and other bibliometric indicators provides useful insight into the validity of these bibliometric measures as surrogates for the measurement of research quality. This is relevant to the debate on the adoption of more metrics-based approaches for research assessment – such as is being proposed in the UK Research Excellence Framework (REF) (successor to the Research Assessment Exercise (RAE)).

## Materials and Methods

### Accessing the cohort

Using the search criterion **Wellcome Trust [gr]** on PubMed, details of the first consecutive 1000 papers associated with the Wellcome Trust published between May and September 2005 were downloaded. The full text of these papers was accessed either via the web, where the paper was available in an open or public access journal or featured in a journal to which the Trust has a subscription, or via request (and payment) from the British Library.

The papers were manually scrutinized and those without a biomedical research focus and/or incorrectly linked to the Wellcome Trust - a small number of papers were linked to either ‘*Burroughs-Wellcome*’ or *‘GlaxoWellcome’* - excluded from the analysis. As a result the ‘PubMed 1000’ became 979, comprised of 157 review (16%) and 822 original research (84%) papers.

### Characterising the papers

The journal title and publisher were noted for all 979 papers. For original research papers (n = 822) details of the author number, institutional collaborations and additional/co-funders were abstracted systematically and the journal impact factor of the featuring journal at the time of publication derived.

A detailed analysis of the nature of the association to the Wellcome Trust was conducted for each original research paper. This was manual and labour intensive as more than two-fifths of original research papers (n = 327/822), other than acknowledging the Wellcome Trust, did not provide any further information on their association (e.g. grant number, author affiliation). Even where there was some indication of the nature of the link to the Wellcome Trust, much of the detail required for this project - such as grant type – was not immediately obvious. As a result, and for each paper, a combination of the information contained in the acknowledgment section, author name/s and institutional address/es and affiliation/s were cross-checked against the Wellcome Trust’s grant database. In many cases, several Wellcome Trust grants were associated with each paper. To simplify the analysis, a maximum of four grants were linked to each paper - those deemed most relevant to the research being chosen.

Papers were classified into broad scientific areas covering: immunology and infectious diseases; molecular and cellular biology; genetics; basic and cognitive neuroscience and mental health; physiological sciences; and epidemiology and public health.

### Wellcome Trust reviewers

An expert Review ‘College’ with relevant scientific expertise, comprising 16 reviewers drawn from senior Trust scientific staff and scientific leaders involved in the Trust’s funding committees, was convened. Reviewers were paired and assigned papers covering their broad scientific expertise. Each reviewer was required to independently read their assigned papers and assess the importance of each according to one of four, semantically-differentiated, categories:

- ‘Landmark’ (assigned a score = 4)
- ‘Major addition to knowledge’ (score = 3)
- ‘Useful step forward’ (score = 2)
- ‘For the record’ (score = 1).

Reviewing was undertaken during December 2005; given that the papers in the cohort were published between May and September 2005, each paper had been published for a maximum of 6 months at the time of its review. The journal in which each paper appeared was not masked; reviewers were simply instructed to assess the research paper itself. While there is evidence that knowledge of author/s, institutional affiliation and featuring journal can effect assessments of published outputs [5], in reality such ‘biases’ are inherent throughout peer review and are difficult to completely counter.

Two assessments were provided for 87% (n = 716/822) of the papers. For 106 papers, two assessments were not provided due to either a conflict of interest for the reviewer and/or the paper being outside the reviewer's area of expertise. Where two reviews were provided, assessments of importance matched exactly, or were one category apart, on 96% of papers (n = 687/716). Where assessments were more than one category apart, the assessment for that paper was ‘unresolved’ and excluded from this initial analysis (n = 29/716). Thus 687 papers received a ‘complete review’ and were included in the subsequent analysis. A simple scoring system was devised to reflect the importance rating of each paper – the score assigned to each paper being the sum of the two scores of the reviewers, ranging from ‘2’ (where both reviewers assigned a score of 1 - ‘for the record’) to a maximum of 8 (where both reviewers assigned a score of 4 - ‘landmark’ paper).

We calculated the weighted kappa statistic to indicate the level of agreement on ‘importance’ between reviewers across all reviewed papers. The weighted kappa statistic overall was 0.132 (‘slight’), indicating that reviewers were likely to agree in their assignment of importance more than would have occurred by chance.

### Tracking & analysing the performance of the papers

After 3 years, ‘performance’ data on all papers was compiled by using the Scopus (citations) and the F1000 (F1000 score and assessment) databases.

As patterns of reviewers' ratings and citations were not normally distributed, non-parametric tests were used to derive levels of statistical significance. Spearman's Rank Correlation (r_s_) was used to measure the level and the statistical significance of the association between reviewers' ratings and other measures of paper performance. The Mann-Whitney U test was used to test the statistical significance of the differences in citation volume of papers associated with three major funding mechanisms (programme and project grants and fellowships).

## Results

The cohort of 979 papers (822 original research and 157 review papers) appeared across 432 different journals, published by 98 different publishers. Original research papers (n = 822) were not concentrated in specific journals; the Journal of Biological Chemistry, featured the highest number of papers (n = 22) and only 11 journals featured more than 10 papers. Seventy per cent of original research papers (n = 573/822) appeared in a journal with a Journal Impact Factor (JIF) above 3.

A large proportion of the papers resulted from collaborative work involving multiple research institutions, often in several countries; less than half (47%) of original research papers were generated solely from UK-based researchers. Nearly two-thirds (64% n = 524/822) of original research papers listed five or more authors. The original research papers were linked to over 900 different Wellcome Trust grants – though more than one Wellcome Trust grant was often acknowledged on papers, alongside a range of other funders. Less than a quarter (23% n = 189/822) of original research papers were linked solely to Wellcome Trust support.

**Figure 1:**
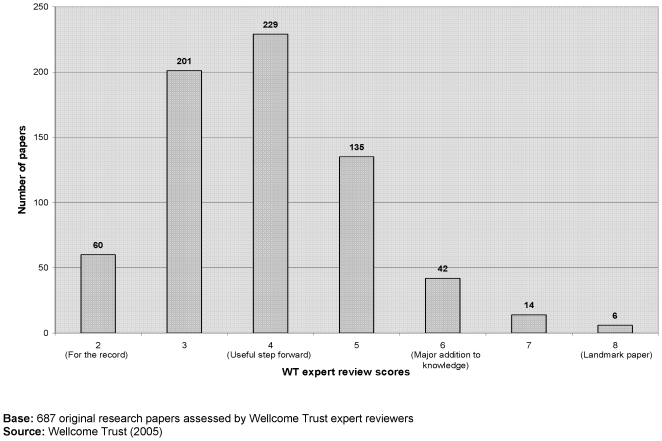
‘PubMed 1000’ original research papers – ‘importance rating’.

**Figure 2:**
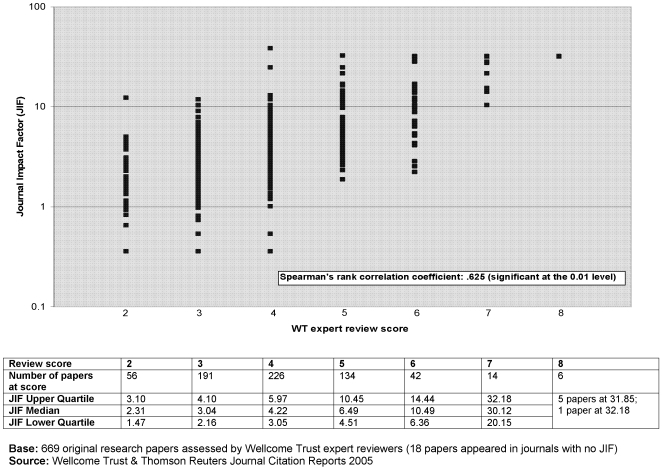
‘PubMed 1000’ – ‘importance rating’ & Journal Impact Factor (2005).

At the time of publication, nine per cent (n = 62/687) of original research papers were considered to describe at least a ‘major addition to knowledge’ (**Figure 1**); six were considered to be ‘landmark’ papers – five of which appeared in *Science* and one in *Nature*, all with an international health focus. Papers were most commonly thought to describe a ‘useful step forward’, with a third receiving this rating (33% n = 229/687). There was a strong positive correlation (Spearman's coefficient, r_s_ = 0.625, significant at 0.01) between the ‘importance rating’ assigned by our experts and the Journal Impact Factor of the featuring journal (**Figure 2**).

**Figure 3:**
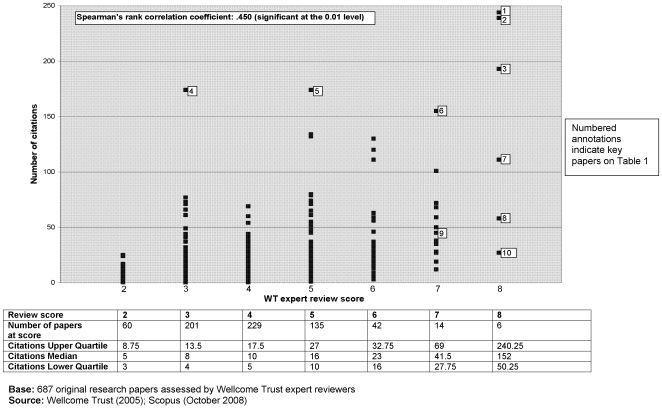
‘PubMed 1000’ - ‘importance rating’ (2005) & citations (2008).

**Figure 4:**
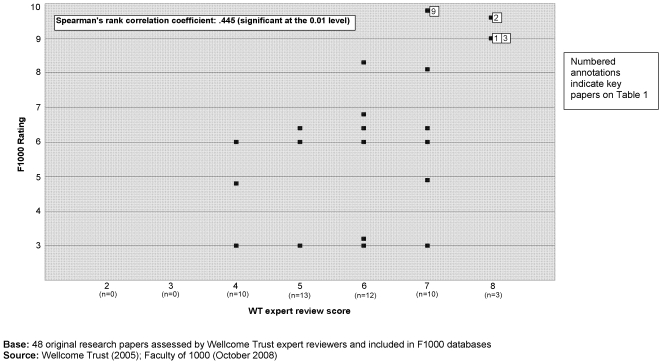
‘PubMed 1000’ – ‘importance rating’ (2005) & F1000 rating (2008).

By the beginning of October 2008 the papers in our cohort had been published for 3 years. Overall papers received an average of 19.48 citations and a median of 12; only 9 papers were not cited at all. There was a positive correlation (r_s_ = 0.45, significant at 0.01) between our reviewers' assessments of the ‘importance’ of the research papers (as reviewed in 2005) and the papers' use in the wider community as indicated by citation totals three years later (**Figure 3**). By the beginning of October 2008, 48 (7%) of the 687 original research papers assessed by our reviewers also featured on the two F1000 databases. Our expert review scores were positively correlated (r_s_ = 0.445, significant at 0.01) with the assessments of these same papers on F1000 (**Figure 4**).

**Figure 5:**
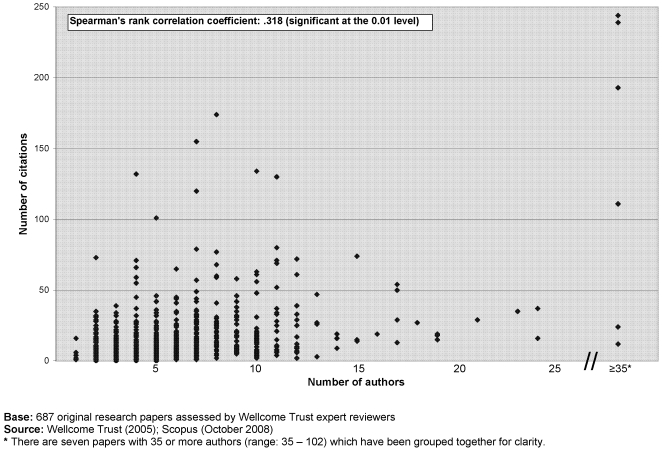
‘PubMed 1000’ – number of authors & citations (2008).

Details of the papers in the cohort receiving the highest scores by our expert reviewers (review score 8; n = 6) and F1000 reviewers (9.8, n = 1) and three other papers achieving among the highest volume of citations after 3 years, are listed in **Table 1**. All 10 of these papers describe a ‘new finding’, though the nature of that ‘finding’ varies from a new genome sequence to new epidemiological data. The top 3 most highly cited papers were genomics-based and had over 80 authors. There is a significant positive correlation between the number of authors and the number of citations a paper has received (**Figure 5**).

**Table 1:** Highly cited & highly reviewed original research papers (October 2005 & 2008).

Key	Original research paper	Citations (Oct 08)	WT Expert review score (max = 8) (2005)	F1000 Rating (Oct 08)	Reason for F1000
1	Ivens AC et al. *The genome of the kinetoplastid parasite, Leishmania major*. (2005) **Science** 309 (5733): 436–42	244	**8**	9	New finding
2	Berriman M et al. *The genome of the African trypanosome Trypanosoma brucei*. (2005) **Science** 309 (5733): 416–22	239	**8**	9.6	New finding
3	El-Sayed NM et al. *The genome sequence of Trypanosoma cruzi, etiologic agent of Chagas disease*. (2005) **Science** 309 (5733): 409–15	193	**8**	9	New finding
4	Hawley SA. et al. *Calmodulin-dependent protein kinase kinase-beta is an alternative upstream kinase for AMP-activated protein kinase*. (2005) **Cell Metabolism** 2 (1): 9–19	174	3	None	N/A
5	Reilly JJ et al. *Early life risk factors for obesity in childhood: cohort study*. (2005) **BMJ** 330 (7504): 1357–1359	174	5	None	N/A
6	LaCava J et al. *RNA degradation by the exosome is promoted by a nuclear polyadenylation complex*. (2005) **Cell** 121 (5): 713–24	155	7	None	N/A
7	El-Sayed NM et al. *Comparative genomics of trypanosomatid parasitic protozoa*. (2005) **Science** 309 (5733): 404–9	111	**8**	None	N/A
8	Carulla N et al. *Molecular recycling within amyloid fibrils*. (2005) **Nature** 436 (7050): 554–8	58	**8**	None	N/A
9	Cliffe LJ et al. *Accelerated intestinal epithelial cell turnover: A new mechanism of parasite expulsion*. (2005) **Science** 308 (5727): 1463–1465	45	7	9.8	New finding
10	Perez-Morga D et al. *Apolipoprotein L-I promotes trypanosome lysis by forming pores in lysosomal membranes*. (2005) **Science** 309 (5733): 469–72	27	**8**	None	N/A

**Source**: Wellcome Trust expert review (2005); F1000 & Scopus (2008).

**Note**: ‘key’ number represents annotation on Figures 3 & 4.

**Figures 3** and **4** are annotated to show the position of these papers in relation to the various measurement criteria. Despite the significant positive correlations between assessments of importance and citations *overall*, at the *individual paper level* the analysis showed that there are exceptions; papers that were highly rated by expert reviewers were not always the most highly cited, and *vice versa*. Additionally, what was highly rated by one set of expert reviewers may not be so by another set; only three of the six ‘landmark’ papers identified by our expert reviewers are currently recommended on the F1000 databases.

One of the core aims of the project was to explore the relative importance and performance of publication outputs associated with different funding mechanisms. In terms of peer assessments of ‘importance’ and citation volume in the three years since publication, there are indications that papers associated with larger awards and training awards (programmes and fellowships (excluding PhD studentships)) performed ‘better’ than papers associated with shorter-term, smaller value awards (**Table 2**); there is a statistically significant difference in the volume of citations emerging from papers linked to programmes and fellowships compared with those linked to projects (programmes compared with projects – Mann-Whitney p = 0.04; fellowships compared with projects – Mann-Whitney p = <0.001)), **Table 2**). As this may be in part a reflection of the size and constituency of the team working on certain grants - for example it is likely that larger programme grants and fellowships involve many, often senior, researchers - research papers associated with such grants may be more likely to have a larger number of associated authors and potentially achieve higher citations rates both through the involvement of ‘senior’ scientists and by virtue of the self-citations linked to the further research of all members of the team. However, this analysis of grant type should be treated tentatively as the analysis was based on a cohort of *papers* published over a specific period and not the total complement of outputs arising from a cohort of *grants*, nor did we explore in detail the nature of other, non-Wellcome Trust funding acknowledged on each paper. As a key strategic issue for research funders, more detailed analyses on the quality and merit of outputs associated with different funding mechanisms are required.

**Table 2:** Original research papers (published 2005) linked to Programme, Fellowship and Project grants & citations per papers (2008).

Grant type	Number of papers linked to grant type*	Min cites/paper	Max cites/paper	Mean cites/paper	Median cites/paper	Inter-quartile (range) cites/paper
Programmes	181	0	244	23.07	13	5.5–23.5
Projects	279	0	244	14.66	9	5–18
Fellowships	214	0	239	22.16	14	7–26

**Base**: 558 original research papers linked to Programme, Fellowship (excluding PhD training studentships) and Project grants and assessed by Wellcome Trust reviewers.

* many papers linked to more than one grant.

**Source**: Wellcome Trust, PubMed (2005) & Scopus (October 2008)

**Note**: Mann-Whitney tests show no statistically significant difference between the citation volume of papers linked to Programmes and Fellowships (Mann-Whitney p = 0.458), but significant differences between the citation volume of papers linked to Programmes, Fellowships and Projects: Programmes and Projects (Mann-Whitney p = 0.04); Fellowships and Projects (Mann-Whitney p = <0.001).

## Discussion

In response to the project aims, overall the different quantitative (journal impact factors and citations) and qualitative (expert review and F1000) analyses provide a relatively consistent assessment of our cohort of papers. In addition, expert reviewers were broadly able to predict the most ‘important’ papers, subsequently identified by another set of experts (F1000) and in terms of their usage in the scientific community, as defined by citations.

Historically, and taken at an aggregate level, bibliometric measures have been used as a proxy indicator of research quality, and particularly in areas, such as the biosciences, where publication output remains a key indicator of research progression. Indeed, this provides much of the rationale for the move to replace the UK’s Research Assessment Exercise (RAE) with a more metric-based successor [6], [7]. However, changes in the nature of science and research, and specifically within the biosciences, make the interpretation of bibliometric analysis increasingly complex.

Scientific research is increasingly a collaborative, multi-disciplinary, multi-location and multi-funded activity [8], [9]; upward trends in paper author number are, at least in part, a reflection of this. While this upward trend is also thought to be linked to research assessment exercises such as the UK RAE [10], [11], it is also likely to continue where there is value in ‘big’, collaborative, international science, such as in the area of genomics. This in turn is likely to contribute to the higher author numbers and citation rates typically found in genomics-based papers but also across other areas. In addition, many scientific papers describing a new technique or dataset with immediate utility will be important immediately and highly cited; other papers, often those describing a new insight to a specific field, will be ‘slow burners’ taking time to gain acceptance and impact [12]. The distinct and different patterns of publication and citation behaviour across areas of science are becoming better understood as bibliometricians develop more insightful methods to accommodate and explain these [13]. The proliferation in the availability of research findings in online, open and public access journals and repositories is also changing the nature of access to research; potentially impacting upon the usage and subsequent citation of research. Add to this recent evidence that increasing access to journals and information online may actually lead to a *reduction* in the number of journals and citations as scientists more quickly tap the consensus of opinion and build their research on this [14] and we find ourselves in a situation where the interpretation of bibliometric analysis as applied to science – and across different fields within science – has become extremely complex.

In terms of more qualitative assessments of paper ‘importance’, we found a good correlation between expert opinion and subsequent ‘performance’ according to quantitative indicators. However, as in traditional peer review and in existing studies correlating expert assessments of the scientific value of a paper with metrics [15], we found substantial variation in perceptions at the level of individual papers. It can also be difficult to anticipate the potential importance of a particular line of research at the time of publication; for much basic, foundation-laying research it takes time for its value to become evident [16], [17]. An increasing number of journals, particularly those based online, have introduced features to enable reader ratings and encourage critique and ‘blogs’ of published research papers. While these can be useful, it can be hard to determine the ‘expertise’ of the reviewer or commentator through these more informal mechanisms of feedback.

We found a highly significant correlation between the importance of papers identified by our expert panel and those identified by the Faculty of 1000 experts. However, only 25 of the 62 papers characterised by our expert panel as being a ‘major addition to knowledge’ or a ‘landmark’ paper were identified by the Faculty of 1000. At least part of the explanation for this discrepancy is that Faculty of 1000 makes no claims to be systematic in its survey of the biomedical literature. Only publications that form part of the regular scrutiny of the literature by the Faculty are screened and a large part of the literature is never assessed by Faculty of 1000 reviewers. These data do support the concept that mechanisms such as Faculty of 1000 of post-publication peer review are a valuable additional mechanism for assessment of the quality of biomedical research literature. Indeed, the data from the present study show that we may be able to address some of the complexity in interpreting bibliometric data combined with the inherent subjectivity of expert review by *linking* qualitative assessments of paper impact and importance with more quantitative assessments.

This work was enabled by our arrangement with the U.S. National Library of Medicine, which systematically identified research papers acknowledging Wellcome Trust funding. However, inconsistencies in acknowledgment practice among authors and journals meant that some papers containing work funded by the Wellcome Trust would have been missed. It is also possible that ‘poorer’ quality papers may also be those providing less complete acknowledgment information though there is no evidence to suggest that this has introduced any systematic bias into this study.

Recent initiatives led by the NIH, the Wellcome Trust and other UKPMC funders, to mandate researchers who receive their funds to deposit papers in open access repositories, should also help funders to gain greater understanding of and access to research outputs associated with their funding support [18]. Furthermore, in recognition of the value of the acknowledgment section of a research paper specifically, there have been several recent initiatives to improve their accessibility and use. For example, the acknowledgment section of papers featuring in UKPMC are now fully searchable, and in January 2009, Thomson Reuters introduced a facility to view and search acknowledgment information on papers held in the Web of Science database. There is also a body of research that has explored the options for measuring scientific contributions through more automatic acknowledgment indexing [19] and initiatives led by several publishers and editors [20]. However, any ‘top down’ developments to improve access to information in the acknowledgment section via bibliographic databases will be largely redundant if researchers do not acknowledge their funding source systematically in the first place.

Led by the Research Information Network in the UK, working with a number of research funders and publishers, a set of simplified and standardised acknowledgment guidelines for researchers has recently been introduced [21]. The guidelines include a set of recommendations to publishers on how to code information contained in an acknowledgment section and has also recommended that publishers include a specific ‘funding’ section on papers. Over time, the combination of ‘top down’ and ‘bottom up’ approaches should improve access and add value to information on published papers associated with research funding and affiliation.

We were also interested to ask whether different types of funding were associated with different qualities of output. We stratified the cohort of publications funded by the Wellcome Trust as project grants (typically three years funding for one or two posts plus running costs), programme grants (typically five years funding for four to six posts plus running costs) or fellowships (typically salary support for the principal investigator for three to five years associated with running costs and variable numbers of additional posts). The outputs linked to project grants were cited significantly less frequently than those linked to programme and fellowship grants. This is a potentially important finding that we will dissect further in future studies. In particular we intend to conduct more detailed analyses of patterns of publication output, subsequent citation and the lag time between funding and ‘impact’, in relation to different grant types, the demographic characteristics of those working on the grants, and the field of scientific research.

For all those that fund research, it is the products of the grant funding that matter. Greater insight into the relative strengths of different modes of funding research in enabling the production of original and important published output would be a valuable input to strategic decision-making for all those involved in supporting research.
